# Acute Respiratory Distress Syndrome and Hepatotoxicity Associated with Single Dose Nitrofurantoin Use

**DOI:** 10.1155/2012/465389

**Published:** 2012-11-27

**Authors:** Gokhan Sargın, Osman Elbek, Cem Balantekin, İbrahim Meteoglu, Nil Culhacı

**Affiliations:** ^1^Department of Internal Medicine, Adnan Menderes University Medical Faculty, Aydin, Turkey; ^2^Department of Chest Diseases, Adnan Menderes University Medical Faculty, Aydin, Turkey; ^3^Division of Gastroenterology, Adnan Menderes University Medical Faculty, Aydin, Turkey; ^4^Department of Pathology, Adnan Menderes University Medical Faculty, Aydin, Turkey

## Abstract

Nitrofurantoin is a synthetic nitrofuran compound. It is generally used in urinary tract infections, either alone or in combination with other antibiotics. A number of adverse effects may develop in different body systems during nitrofurantoin treatment; however, concomitant pulmonary and hepatotoxicity are rare. In this paper, we present a case of acute respiratory distress syndrome and hepatotoxicity following administration of a single dose of nitrofurantoin.

## 1. Introduction

Nitrofurantoin (NF) inhibits carbohydrate metabolism and many bacterial enzymes involved in synthesis of DNA/RNA; it is used in uncomplicated urinary tract infections or to prevent recurrence of chronic urinary tract infection [1]. It is known that NF may cause pulmonary reactions and hepatotoxicity [1–4]. Nevertheless, simultaneous development of pathological changes in the lung and liver is a very rare occasion [2]. On the other hand, the literature search did not reveal any articles reporting cases of concurrent development of acute respiratory distress syndrome (ARDS) and hepatotoxicity with administration of a single dose of this agent.

## 2. Case Presentation

Forty-one-year-old female patient referred to our hospital with nausea, epigastric tenderness, and cough. She had a confirmed diagnosis of type 2 diabetes mellitus and informed that one dose 100 mg NF was administered four days ago for urinary tract infection. Her general condition was moderate, blood pressure 120/80 mmHg, pulse 94/minute, respiratory rate 36/minute, fever 37.8°C, and oxygen saturation was 89%. Physical examination revealed bilateral crackles in the lungs and liver was palpable about 3 cm below the right costal margin in midclavicular line. 

Patient's laboratory test results were as follows: hematocrit 28.8%, white blood cells 8.58 × 10^3^/microL (neutrophils: 5.48 × 10^3^/microL, eosinophils: 0.07 × 10^3^/microL), platelet count 297 × 10^3^/microL, alkaline phosphatase 255 U/L, gamma glutamyl transferase 250 U/L, and sedimentation 100 mm/hour. Protein (+1) and leukocyte (+3) were detected in urine sample. Moderate hypoxemia was detected in arterial blood gas analysis and P_a_O_2_/F_i_O_2_ was 136 mmHg. Serological examination showed 1/1000 positive antinuclear antibodies (ANA) with centromeric positive pattern and viral and other serologic markers were negative.

**Figure 1:**
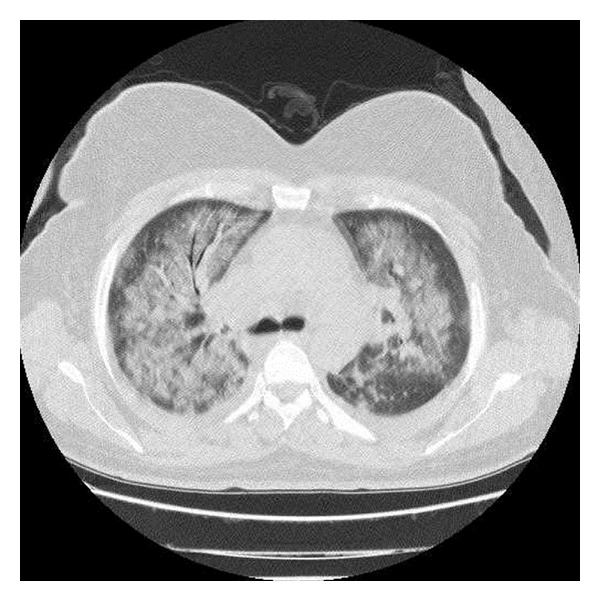
High resolution computed tomography revealed extensive ground-glass infiltrates in both lungs.

The patient had a normal chest radiograph prior to administration of nitrofurantoin; however, chest radiograph at the time of referral to our hospital revealed bilateral and diffuse interstitial densities. High Resolution Computed Tomography (HRCT) revealed extensive ground-glass infiltrates in both lungs (Figure 1) and abdominal ultrasound examination revealed hepatosplenomegaly, hepatosteatosis, and liver with heterogenous parenchyma.

**Figure 2:**
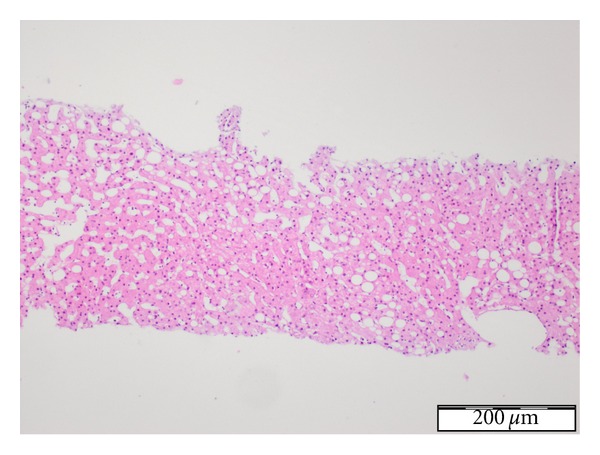
Transbronchial biopsy specimen showed mild interstitial fibrosis and inflammation.

**Figure 3:**
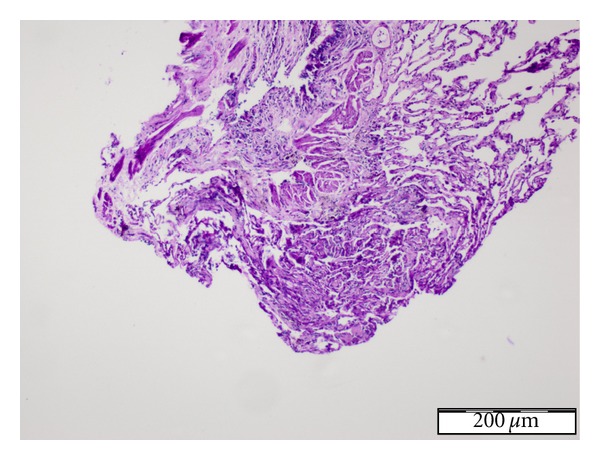
Hepatic parenchymal inflammatory cell infiltrates and sinusoidal dilatation in liver biopsy specimens.

Based on clinical and laboratory findings, the case was diagnosed as acute lung injury (ALI) and hepatotoxicity secondary to use of NF. Alveolar hemorrhagia was excluded by negative findings in bronchoalveolar lavage. Transbronchial biopsy specimen showed mild interstitial fibrosis and inflammation (Figure 2). There were hepatic parenchymal inflammatory cell infiltrates and sinusoidal dilatation in liver biopsy specimens (Figure 3). Upon progression of clinical state to ARDS within 48 hours despite discontinuation of nitrofurantoin and supportive treatment with 2 L/min oxygen, pulsed steroids were used for 3 days, followed by 1 mg/kg/day methylprednisolone, administered concurrently with non-invasive mechanical ventilation (NIMV). Following steroid treatment, chest radiography and HRCT showed significant regression and improvement in oxygenation. The patient was discharged on the 14th day of hospitalization. Steroid therapy was tapered gradually and discontinued at the end of the 6th week upon normalization of liver function tests.

## 3. Discussion

In this paper, we presented a case of concurrent ARDS and toxic hepatitis, which developed with administration of a single dose of NF and fully recovered with steroid therapy and NIMV.

NF is absorbed from the gastrointestinal tract, metabolized by glutathione S-reductase enzyme system in liver, and is mainly excreted by urinary system [1, 2].

NF is one of the several agents that may cause concomitant pulmonary and hepatotoxicity, as in the case presented in this paper. Similarly, busulfan, chlorambucil, amiodarone, and methotrexate may also cause concurrent pathological changes in lung and liver [5]. However, there are no cases in the literature reporting development of toxic hepatitis and ARDS with a single dose of this agent, as seen in our case.

NF is generally used in treatment of urinary tract infections and the most common side effects associated with the agent are designated as nausea, bloating, and headache. In rare cases, serious side effects such as pulmonary and hepatic toxicity may be observed in patients receiving prophylactic treatment with long-term and low dose NF [1–5]. Due to higher rate of concomitant autoimmune diseases among women, it is well known that women suffer side effects of NF more often than men [6]. The fact that our case was a female patient with positive ANA and pathological changes in lung and liver developing after administration of single dose NF suggest that NF-associated toxicity is based on immune system.

Indeed, development of NF-associated toxicity is independent of the dose and it is observed in relatively low dose ranges [7]. Koulaouzidis et al. reported a case of pulmonary and hepatotoxicity with the use of low dose (100 mg daily) NF for 16 months [2]. Similarly, isolated acute respiratory failure developed in a pregnant patient after 48 hours with the use of NF (200 mg daily) [7]. Holmberg and Boman reported that acute lung injury symptoms associated with use of NF developed after long duration such as 2-3 weeks [8]. In our case, ALI occurred after 24 hours and ARDS developed after 72 hours following administration of 100 mg NF.

NF-induced pulmonary reactions have been reported since 1959. Pulmonary pathologies associated with NF are bronchiolitis obliterans organizing pneumonia, diffuse alveolar hemorrhagia, diffuse alveolar damage, and acute, subacute, and chronic interstitial lung disease [8]. Clinical manifestations are usually associated with acute hypersensitivity reaction due to type I and III immune reactions. Consolidation or pleural effusion at baseline may be seen on chest X-ray. However, radiological findings including diffuse fibrotic changes rapidly disappear upon discontinuation of the drug [2, 4, 9]. In our case, alveolar hemorrhage was excluded by examination of bronchoalveolar lavage and presence of pulmonary parenchymal fibrosis was shown by transbronchial biopsy. However, unlike the acute clinical manifestations reported in the literature, clinical status and radiological changes worsened after withdrawal of the drug and ARDS developed in our patient within 72 hours.

On the other hand, NF may also cause elevated liver enzymes, severe symptomatic chronic active hepatitis, and, even, liver failure leading to liver transplantation [2]. Determination of drug-induced liver toxicity is complex and biopsy may be required. Drug may cause damage of hepatocytes by direct effect with oxidative stress or through provision of HLA1 to hepatocyte membrane by the drug itself or breakdown products, thus activating cytotoxic T cells [10, 11]. Alternatively, NF-associated acute hepatitis may develop as a hypersensitivity reaction within several weeks after administration of the agent [11]. However, chronic hepatitis is only observed during long-term use and pathophysiology is described with immunoallergic mechanisms [12]. Female gender, presence of autoantibodies such as ANA and anti-smooth muscle antibody, hypergammaglobulinemia, and histological findings are considered as lines of evidence of immunologic reaction [1, 12, 13]. Similarly, presence of inflammatory cells in liver biopsy and positive ANA found in our patient also suggest that immunological reaction is responsible for the development of clinical symptoms.

The first step in treatment is the withdrawal of the drug. Discontinuation of the drug is generally sufficient for reversal of liver damage. However, steroid treatment may be beneficial in patients with persistent elevation in liver function tests, despite withdrawal of the drug [12]. It is not clear whether steroid therapy exerts a beneficial effect, independent from withdrawal of the agent [7]. Nevertheless, withdrawal of NF alone, without concomitant immunosuppressive drugs, was indicated to be insufficient in patients of hepatotoxicity associated with immunological mechanisms [12]. On the other hand, clinical and radiographic improvement was reported in the literature with the use of corticosteroids in a patient with concurrent pulmonary and liver toxicity [2]. In our patient, clinic status worsened and ARDS developed, despite withdrawal of the drug. ARDS and toxic hepatitis improved with steroid therapy. These findings suggest that discontinuation of the drug alone may not be sufficient in treatment of NF toxicity associated with immunological reactions. 

In conclusion, clinicians should always consider that each therapeutic agent may cause serious side effects, which may lead to mortality. History of drug use should be investigated independent of dose in patients referring with ARDS and/or hepatotoxicity. Steroid therapy should be initiated in patients where the clinical state does not show any improvement despite withdrawal of the drug.
